# An Historical View of Surgery, in the Sixteenth Century

**Published:** 1799-08

**Authors:** 


### *An Historical View of Surgery, in the Sixteenth Century.*

[Extracted from the original German of Professor CURTIUS SPRENGEL'S "*History of Medicine, founded on authentic Documents:*"* Vol. III. Sect. X. p. 457, & seq.]

§ 1. THE art of surgery is unquestionably a branch of medicine, and its vicissitudes must therefore, in every respect, be similar to those changes which medicine has successively undergone: this observation is amply confirmed by the events which took place in the sixteenth century, after the revival of all the sciences. The first surgeons of this century were almost unanimously blind followers of *Abu'l Kasem*, and of *Guy de Chauliac;* they dreaded chirurgical operations, and endeavoured to supply their want of dexterity by increasing the number of cataplasms and ointments. A few of the more expert surgeons occasionally attempted difficult operations, but their learned brethren could not be easily convinced of the advantages attending them. The Gothic taste still prevailed in the construction of chirurgical instruments, which were so complicated and artificial, that the principal difficulties were thus rather aggravated than diminished.

§ 2. Innumerable instances might be produced, from which it would appear, that the learned surgeons of this century very seldom attempted to perform difficult operations; and that these were generally intrusted to ignorant barbers, or itinerant pretenders. A few of the most striking facts will sufficiently illustrate this assertion. When King Matthias, of Hungary, was wounded in a battle against the Moldavians, in 1464, the arrow remained so closely fastened in the wound, that none of the royal surgeons could extract it. The King therefore issued a proclamation, in which he offered riches and honours to the surgeon who would repair to his court, and heal the wound.

Four years elapsed before any adventurer appeared. At length, *John of Dockenburg*, a surgeon from Alsatia, ventured to undertake this task; he travelled to Hungary, saved the King, and was loaded with extraordinary rewards.

The most celebrated physicians and surgeons of Italy, such as *Joh. de Vigo*, *Joh. Bapt. Sylvaticus*, and others, left the great operations of lithotomy, trepanning, and the extraction of the cataract, to itinerant operators; and among these we find an Italian family named *Norsini*, of Milan, particularly celebrated for a century past, on account of their successful operations for the stone. Even in the fifteenth century, an operator of that family made a journey to France, where he taught his art to *Germain Colot*,

* See the Account we have given of this important work in our first Volume, p. 83.

*Colot*, a ſurgeon of that country.—This ingenious pupil impatiently waited for an opportunity of performing his firſt operation. At length, in 1474, ſuch an opportunity occurred, when a criminal of Meudon (others ſay of Bagnolet), for robberies committed in the woods, was condemned to die. Happily for the art of ſurgery, this delinquent was afflicted with the ſtone; but the hiſtorians do not mention whether it was in the kidneys, or in the bladder; it is, however, probable that it was in the latter.

The French ſurgeons repreſented to King Louis XI. that, if this experiment on the criminal were permitted, and ſhould terminate ſucceſsfully, it would be the means of ſaving the lives of many unfortunate individuals, and delivering them from their torments. The King granted the requeſt, and Colot performed this famous operation ſo ſucceſsfully, that the convict was reſtored in a fortnight, and conſequently obtained a full pardon.

But what method the courageous Colot adopted in this operation, we cannot learn from the imperfect accounts given of it by his contemporary writers, *Troyes*, in his "*Cronicle Scandaleuſe*;" *Comines*, in his "*Memoires*;" and *Villaret* and *Garnier*, in their "*Hiſtoire de la France*," vol. xviii. p. 124.—It is, however, reaſonable to ſuppoſe, that he employed the high operation, becauſe he mentions, among other circumſtances, the reduction of the inteſtines, and the ſuture of the abdomen.

*John Lange*, who had ſtudied in Italy, and likewiſe benefited by the inſtructions of *Joh. de Vigo*, declares, that he had never ſeen the trepan applied by this celebrated ſurgeon. On his return to Germany, he cauſed an inſtrument to be made, to which he gave the pompous name of *trepanum abaptiſton*, and exhibited it in an aſſembly of German ſurgeons and phyſicians. Theſe, full of aſtoniſhment, exclaimed, "Langi Doctor! fruſtra quæris in Germania abaptiſta; non enim chirurgorum inſtrumenta nobiſcum, ſed campanæ et pueri baptizantur." One of the company facetiouſly remarked, that even chirurgical inſtruments might, with more propriety, be baptized in Rome, where his holineſs the Pope had fixed his reſidence.

§. 3. Several branches of ſurgery, however, were particularly cultivated, and thus gradually arrived at a more improved ſtate. Of this deſcription is chiefly the treatment of *gun-ſhot wounds*, which could not be derived from the Arabians and Saracens, but which was a new and original effort of this century; and hence the theory and treatment of theſe wounds were liable to many ſubſequent changes.

*Hieron. Braunſchweig*, ſurgeon at Straſburg, towards the end of the fifteenth century, treated gun-ſhot wounds in a manner exactly ſimilar to that of wounds wounds arifing from poifon. He introduced a tent made of bacon into the wound, and adminiftered the theriaca internally, with a view to expel the virus. Joh. de Vigo endeavoured to explain the danger attending gun-fhot wounds, partly from the round figure of the bullet, partly from the (apparent) fearing of the part, which it was fuppofed always took place, and partly from the poifonous qualities of the bullet and the powder.

According to this erroneous opinion, he propofed two diftinct remedies; firft, to moiften the wound, to alleviate the effects of combuftion; and fecond, to dry, and, as it were, exficcate the virus. Vigo previoufly cauterifed the gun-fhot wound with an intention to deftroy the poifon; or he applied the Egyptian ointment, and fometimes very hot oil. After this he directed a liniment of frefh butter, in order to refolve the fcab formed over the wound; he alfo recommends a digeftive ointment, compofed of the oil of turpentine, and the yolk of eggs, to affuage the pain.

*Alphonf. Ferri*, of Faenza, formerly furgeon at Naples, and afterwards phyficician to Pope Paul III. likewife maintained, that gun-fhot wounds were of a poifonous nature; an opinion which he had conceived from having obferved the fudden effect of contufion produced by the paffage of a bullet through the air, which frequently occafions fudden death, and which, in its fatal effects, is fimilar to mephitic vapours. He likewife treated gun-fhot wounds with a cauftic of his own invention, which confifted of corrofive fublimate, vitriol, and litharge. Although he was the firft who confidered the extraction of the bullet as indifpenfable to the cure of gun-fhot wounds, yet he did not enlarge the wound at all, but extracted the bullet with an awkwardly-contrived inftrument, which he called *Alfonfinum*. He further maintained, that the bullet may even be left in the body, without danger, as there are examples that it has remained there for twenty years, without detriment. His advice of keeping gun-fhot wounds very clean is little to the purpofe, as it obvioufly proves that he did not underftand the diftinction between pus and fanies.

By the endeavours of *Paré* and *Maggi*, the theory as well as the treatment of gun-fhot wounds were much improved. It is uncertain which of thefe two reformers we are principally indebted for thefe improvements. *Paré* himfelf owns that he is under very great obligations to the Italian furgeons, and hence it has been juftly conjectured that Maggi muft have been his teacher.

It was this ingenious practitioner who, by actual experiments, fucceeded in controverting the erroneous opinion, that gun-fhot wounds are connected with combuftion: "The bullets," fays he, "are not hot when expelled from from the barrel of a gun; they do not set the wadding on fire, and hence, their burning effect is merely chimerical. Nor is there any poison in gunpowder, as none of its constituent parts possess a poisonous property." Both the theory and practice relative to gun-shot wounds, have been considerably improved by this excellent surgeon: he extended the wound by *dilatatoria*, usually prepared of the gentian-root, and he carefully extracted bullets, as well as small shot.

He generally applied mild remedies, especially the oil of roses; he severely censured the frequent cleansing of wounds; and if bones be fractured, he does not advise to extract every splinter, but rather to apply the expulsive bandage. Maggi likewise recommended amputation in all those cases where sphacelus is the consequence of a wounded artery. In such a case, he made the incision in the healthy part, and caused parts of the integuments to hang down over the cutaneous muscles, in order to cover the stump with them afterwards.

§. 4. Paré endeavoured to introduce in France the treatment of gun-shot wounds, established by Maggi, in Italy. He, at the same time, confuted the opinion of the poisonous nature of gun-shot wounds, together with the idea that they ought to be treated in a manner similar to burns; while he deprecated the use of hot oil, formerly recommended by Vigo, and proposed, in its place, the very opposite, namely, the suppurating remedies prescribed by Maggi. In hydropic swellings, that frequently accompany gun-shot wounds, Paré particularly recommends the expulsive ligature, and also the Egyptian ointment. A celebrated Italian surgeon, *Joh. Bapt. Carcano Leone*, of Milan, Professor, in Pavia, likewise defended the theory maintained by Maggi.

*Botalli*, who wrote a treatise on gun-shot wounds, controverted the opinion, that they should be treated like injuries arising from the external application of poison, or from burns; and he consequently treated them as mere contusions. Above all, he extracted the bullets, but left the splinters of bone in the wound, till they were extruded by the expulsive ligature. In the writings of *Fallopius*, we also find an ample detail of the ideas of Maggi and Paré.—*Felix Würz*, an excellent German surgeon, justly deprecated the numerous artificial instruments then used for the extraction of bullets; he condemned the setons, or strings anointed with lard, to which the pretended poisonous matter was supposed to adhere, and censured the use of all fat substances and ointments for burns in the cure of gun-shot wounds. He observed in every respect the antiphlogistic regimen, and applied externally honey, and dilatatoria made of gum tragacanth.—We meet with similar principles in *Guillemeau*, who insists particularly on the dilatation of the wound, and the speedy extraction of the bullet.—*Fran. Ranchin*, chancellor, at Montpellier, modified, in a slight degree, the idea conceived by Botalli; since he considered gun-shot wounds as simple wounds which are *complicated* with contusions, and contended that they were not to be treated as *simple* contusions.

§. 5. The induration of the prostrate gland; the warts in the urethra; and the application of bougies against this complaint, excited great attention about the middle of the sixteenth century, particularly in Spain.—*Philipp*, a surgeon, of Lisbon, pretended to be the inventor of bougies, and travelled through all Europe, with a view to acquire riches by their application. Indeed, *Franc. Diaz*, Professor at Alcala de Henares, seriously considers him as the inventor, but, improperly calls *Philipp* an apothecary, and farther informs us, that a Portuguese merchant, *Alonzo Diaz*, had likewise travelled in the character of a charlatan, and applied these bougies under the assumed name of *Romano*. Yet this assertion was contradicted by *Amatus*, of Portugal, who assures us, that he had been well acquainted with surgeon Philipp, whom he had taught the application of bougies in Lisbon, in 1541, being the same year in which the Emperor undertook the expedition against Tunis; and for the truth of this fact, he produces the evidence of three Portuguese contemporaries: at the same time he ingenuously confesses that he himself is indebted for the knowledge of this remedy to his teacher, Prof. *Aldarette*.

This narrative possesses the greatest share of probability, so that Amatus ought, at least, to be considered as the chief promoter of this remedy.

*Andr. Laguna*, of Segovia, a learned, and very experienced physician, who, in the Spanish war in Flanders, and other campaigns, had made many valuable observations, was one of the first who wrote on that disease, and its new remedy, a treatise entitled "*Andr. Lacunæ methodus cognoscendi et extirpandi excrescentes in vesicæ collo carunculas*," 12mo. Romæ, 1551. *Portal*, the French medical historian, however, is much mistaken, when he maintains that this essay of Laguna appeared so early as the year 1535. Nor was the work of another writer on the same subject, "*Ferrus de caruncula in Uffenbach, thesaur. chirurg.*" published earlier than the year 1551. The latter ascribes the induration of the prostrate gland, to the deposition or collection of mucus, suppuration, and gonorrhœa; he employs first emollients in injections, afterwards bougies with verdigrease, frequently even arsenic with unslacked lime, and lastly healing and cicatrizing remedies.

*Christ. de Vega*, in his work, "*De curatione caruncularum*," 4to. Salamant. 1552,

1552, generally follows the prescriptions of Ferri; but *Amatus Lusitanus*, in his "*Curat. medecin. cent.*" iv. cur. 19, p. 337, properly limits and defines the cases in which strong caustics might be applied, and seriously speaks of the fatal consequences attending the use of white lead, which Laguna had recommended for injections.

*Franc. Diaz*, in his treatise on the same subject, published at Madrid, in 1588, in the Spanish language, likewise advises the indiscriminate use of caustics, and the uninterrupted application of bougies, in order to prevent any new concretions: and in cases where the usual *specillum cereum*, or wax taper, was insufficient, he recommends the use of leaden rods, or triangular needles, for the extirpation of warts.

§. 6. The doctrine of *lithotomy* was considerably improved in this century, by the invention of two different methods of operating, namely, the *great*, and the *high operation*. It has already been mentioned, that Germain Colot undertook a succesful operation for the stone, in the fifteenth century, and probably by the high operation: but it does not appear that learned surgeons had imitated this method, till an obscure practitioner at Cremona, *Joh. de Romani*, in 1525, began to adopt what is commonly called the high operation: he taught it to *Mariano Santo de Barletta*, a surgeon at Naples, who described the particulars of it in a separate treatise, published at Venice, in 1543, wherein he professes to have been a pupil of Joh. de Romani. It is probable that previous to this time no other method of operating was practised than that known under the name of the smaller apparatus, which can be employed only on children under fourteen years of age. In some rare instances which are related by *Benivieni* and *Christ. de Vega*, particularly in women, the stone had been found in the urethra itself, in which cases it could be more easily extracted. But, since that period, the passage was cleared by the application of the gorget, by means of which the forceps could be introduced into the bladder. *Mariano Santo* made use of the following apparatus: he first employed a curved sound, which he introduced into the urethra so as to direct the point to the left side; he expresly cautioned the operator against the incision into the perinæum, and is therefore unjustly censured for having attempted the incision in the middle. His sound was excavated, and he performed the incision in the direction of the groove; then introduced the sound, and along with it the conductors, and afterwards the gorget, which, according to its original construction, terminated in a blunt point; and lastly, he extracted the stone with the forceps, and removed the remaining particles of it, as well as the gravel or sand, by means of the lithotomical spoon. By the application of the blunt dilator, the parts were necessarily lacerated, and the wound occasioned by this laceration could not be healed without great difficulty. Hence *Le Dran* endeavoured to improve upon this method, especially by making an incision through the prostrate gland and the bladder with his guarded knife *(couteau en rondache)*; and the immortal *Schmucker*, of Berlin, was uncommonly successful in using the great apparatus for lithotomy in that improved state.

Mariano Santo communicated his method to *Ottavian de Villa*, who practised surgery at Rome, but afterwards travelled in the character of an operator. Among other countries, he visited France, where he became acquainted with *Laurent Colot* (probably a descendant of Germain Colot), who was instructed by him in his peculiar manner of operating. By his successful operations, Laurent soon acquired great reputation; in consequence of which Henry II. of France called him to his court, and patients from most countries in Europe resorted to Paris, in order to obtain relief from his skilful hand. But our lithotomist carefully concealed his art as a secret, so that it exclusively became the inheritance of his sons. Paré, in his "*Oeuvres*," liv. xx. ch. 8. p. 477, cites two instances, demonstrative of the fortunate success of their operations. Philip Colot, who was either a son or a nephew of Laurent, adopted two assistants, namely, *Severin Pineau*, and *Gyraut*, because he was no longer able to perform the operations. Pineau received orders from the King to instruct ten other surgeons in this useful art; but the royal mandate was not complied with. It is further asserted, that he published a description of his method in a particular work, but it has never come to light. At length, a later descendant of this family, *Franc. Colot*, described the whole of the operation in a pamphlet, entitled "*Traité de l'operation de la taille*," p. 74, et seq.

[To be continued in our next Number.]

